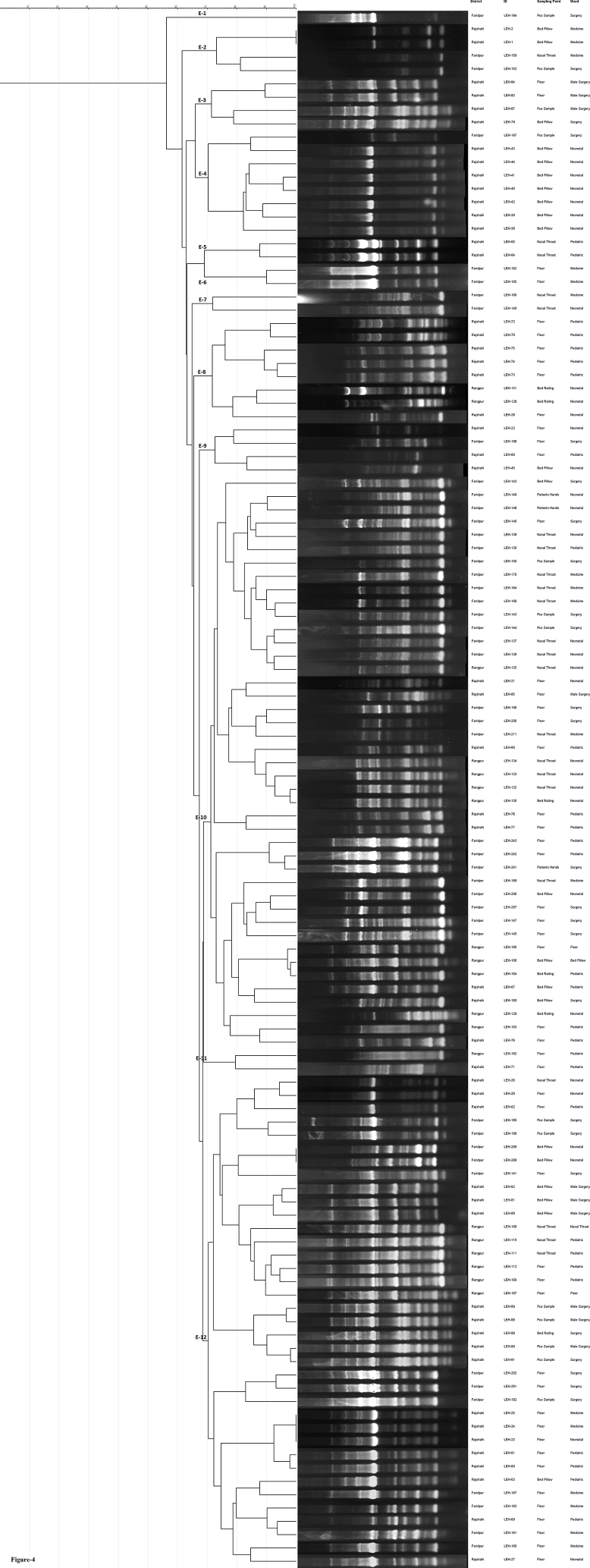# Multidrug-resistant *Escherichia coli* isolated from patients and surrounding hospital environments in Bangladesh: A molecular approach for the determination of pathogenicity and resistance

**DOI:** 10.1016/j.heliyon.2023.e22109

**Published:** 2023-11-07

**Authors:** M. Moniruzzaman, Mohammed Tanveer Hussain, Sobur Ali, Monir Hossain, Md. Sakib Hossain, Mohammad Atique Ul Alam, Faisal Chowdhury Galib, Md. Tamzid Islam, Partha Paul, Md. Shafiqul Islam, Mahbubul H. Siddiqee, Dinesh Mondal, Shahana Parveen, Zahid Hayat Mahmud

**Affiliations:** a Laboratory of Environmental Health, Health Systems and Population Studies Division, International Centre for Diarrhoeal Disease Research, Bangladesh (icddr,b), Dhaka 1212, Bangladesh; b Emerging Infections, Infectious Diseases Division, International Centre for Diarrhoeal Disease Research, Bangladesh (icddr,b), Dhaka 1212, Bangladesh; c Microbiology Program, Department of Mathematics and Natural Sciences, BRAC University, Mohakhali-66, Dhaka, Bangladesh; d BCSIR Rajshahi Laboratories, Bangladesh Council of Scientific and Industrial Research, Dhaka, Bangladesh; e Burnett School of Biomedical Sciences, University of Central Florida, Orlando, FL, USA; f Department of Microbiology and Immunology, SUNY Upstate Medical University, Syracuse, NY 13210, USA; g Department of Biostatistics & Data Science, University of Kansas Medical Center, Kansas City, USA; h Department of Microbiology, University of Manitoba, Winnipeg, MB R3T 2N2, Canada

**Keywords:** Antibiotic resistance, ESBL, *E. coli*, Biofilm, ExPEC, Hospital environments, Whole genome sequencing (WGS)

## Abstract

Extended spectrum β-lactamase producing *Escherichia coli* (ESBL *E. coli*) is a primary concern for hospital and community healthcare settings, often linked to an increased incidence of nosocomial infections. This study investigated the characteristics of ESBL *E. coli* isolated from hospital environments and clinical samples. In total, 117 ESBL *E. coli* isolates were obtained. The isolates were subjected to molecular analysis for the presence of resistance and virulence genes, antibiotic susceptibility testing, quantitative adherence assay, ERIC-PCR for phylogenetic analysis and whole genome sequencing of four highly drug resistant isolates. Out of the 117 isolates, 68.4% were positive for *bla*_CTX-M_, 39.3% for *bla*_TEM_, 30.8% for *bla*_NDM-1_, 13.7% for *bla*_OXA_ and 1.7% for *bla*_SHV_ gene. Upon screening for diarrheagenic genes, no isolates were found to harbour any of the tested genes. In the case of extraintestinal pathogenic *E. coli* (ExPEC) virulence factors, 7.6%, 11%, 5.9%, 4.3% and 21.2% of isolates harbored the *focG, kpsMII, sfaS, afa* and *iutA* genes, respectively. At a temperature of 25°C, 14.5% of isolates exhibited strong biofilm formation with 21.4% and 28.2% exhibiting moderate and weak biofilm formation respectively, whereas 35.9% were non-biofilm formers. On the other hand at 37°C, 2.6% of isolates showed strong biofilm formation with 3.4% and 31.6% showing moderate and weak biofilm formation respectively, whereas, 62.4% were non-biofilm formers. Regarding antibiotic susceptibility testing, all isolates were found to be multidrug-resistant (MDR), with 30 isolates being highly drug resistant. ERIC-PCR resulted in 12 clusters, with cluster E−10 containing the maximum number of isolates. Hierarchical clustering and correlation analysis revealed associations between environmental and clinical isolates, indicating likely transmission and dissemination from the hospital environment to the patients. The whole genome sequencing of four highly drug resistant ExPEC isolates showed the presence of various antimicrobial resistance genes, virulence factors and mobile genetic elements, with isolates harbouring the plasmid incompatibility group IncF (FII, FIB, FIA). The sequenced isolates were identified as human pathogens with a 93.3% average score. This study suggests that ESBL producing *E. coli* are prevalent in the healthcare settings of Bangladesh, acting as a potential reservoir for AMR bacteria. This information may have a profound effect on treatment, and improvements in public healthcare policies are a necessity to combat the increased incidences of hospital-acquired infections in the country.

## 1 Introduction

The continuous and excessive use of antibiotics have led to a global rise in consumption rates. As a result, the levels of antibiotic resistance among pathogenic organisms have increased, leading to a lack of therapeutic options available for treatment [1]. Most national, international, and regulatory agencies consider antimicrobial resistance (AMR) a growing concern. AMR is one of the top ten threats to public health, as listed by the World Health Organization [2]. An estimated 1.27 million deaths resulted from AMR bacteria in 2019, and by 2050 the number is predicted to rise to 10 million deaths a year [3]. Resistance development in bacteria is due to multiple factors, including the indiscriminate use of antibiotics, incomplete treatment completion and inadequate dosage [4]. One of the primary ways in which bacteria gain resistance is through the breakdown of antibiotics utilizing β-lactamases and extended spectrum β-lactamases (ESBLs) [5]. ESBLs are class-A β-lactamases that are able to hydrolyse extended spectrum cephalosporins and monobactams [6]. There are mainly three ESBL enzymes, CTX-M, SHV and TEM, all sharing 25% homology among them [7]. However, unlike the CTX-M groups, which are all classified as ESBLs due to their activity against cefotaxime, only certain variants of the TEM and SHV groups are classified as ESBLs due to their activity spectrum [8]. Most often, the resistance to antibiotics is plasmid-encoded, being disseminated between bacteria through horizontal gene transfer [9]. Through this process, pathogens can acquire genes conferring resistance from environmental bacteria [10].

In 2019, a study found that *E. coli* was the leading pathogenic organism causing deaths associated with and attributable to bacterial antibiotic resistance [3]. Although most *E. coli* strains appear safe, some have disease-causing characteristics that can lead to life-threatening illnesses. There are six distinct well-known pathotypes of diarrheagenic *E. coli* that can infect humans [11] and ExPEC is a term for *E. coli* pathotypes associated with extraintestinal infections [12]. ESBL *E. coli* is a well-known multidrug-resistant bacteria responsible for causing severe hospital and community acquired infections, especially in lower-middle income countries with poor sanitation and inadequate hygiene practices [13]. Compared to non-ESBL *E. coli,* infections by ESBL *E. coli* often cause increased mortality, morbidity, extended hospital stays and healthcare costs [14]. Furthermore, ESBL *E. coli* is resistant to several antibiotic agents that are recommended for treating *E. coli* infections, leading to high chance that the prescribed antibiotics will be ineffective for treatment [7]. Multiple strains of pathogenic *E. coli* are known to be ESBL producers, resulting from horizontal gene transfer of ESBL genes into the existing pathogenic strains. These strains can often emerge within populations through clonal dissemination resulting in high-risk clones [15]. Likewise, transposable elements encoding New Delhi metallo-β-lactamase (NDM) genes have been identified as the cause of rapid spread of carbapenem resistance in *E. coli* [16]. In addition, *E. coli* has been recognized as a well established biofilm former on biotic and abiotic surfaces [17]. The formation of biofilm by *E. coli* has been linked to increased antimicrobial resistance and a variety of nosocomial infections caused due to the colonization of medical devices such as prosthetic joints, grafts, catheters etc [18].

Due to the gradual accumulation of mutation in genomes, strains resistant to multiple antibiotics are emerging at a drastic rate [19]. Consequently, the use of whole genome sequencing (WGS) of these organisms has become an invaluable tool for uncovering mutations, understanding the functions of the mutated genes and allowing genomic characterization of these isolates [20]. The sequencing data can also be used to uncover the presence of particular resistance and virulence genes [21]. Further, WGS also offers various other functions, for example, in the development of new antibiotics, control of antibiotic resistance, enhancement of diagnostics and solving public health problems [22]. WGS enables the in-depth characterization of the bacterium, providing data such as serotype, sequence type (ST), carriage of mobile genetic elements, and presence of antibiotic and virulence determinants, therefore serving as an essential tool for surveillance and outbreak prediction [23].

In Bangladesh, few studies have been conducted on phenotypic and molecular characterization of ESBL *E. coli* with an emphasis on clinical or environmental isolates [13,[24], [25], [26]]. However, they are inadequate given the important roles of hospital environments in transmitting these pathogens. The presence of resistant organisms in hospital environments is a critical source of nosocomial infections. The contamination of intermediate objects is a common transmission source between patients, from healthcare workers to patients and visitors to patients [27]. Additionally, in many healthcare-associated infections, the extent of hospital surface contamination is known to play a role in the risk of transmission [28]. In Bangladesh, recent studies have isolated multidrug-resistant bacteria from hospital environments [29,30]. However, to date, no study has assessed the presence of ESBL *E. coli* within hospital environments in the country.

Therefore, this study investigated the presence of ESBL-producing *E. coli* in hospital environments and clinical samples from three different hospitals in Bangladesh. The isolates were screened for virulence and resistance genes alongside their biofilm-forming properties and antibiotic resistance profiles. Whole genome sequencing of the highly drug resistant pathogenic isolates was conducted to uncover the cause of resistance on a genomic level. Further, pangenome analysis was carried out to reveal the genetic similarities among national isolates sequenced previously.

## 2 Results

### 2.1 High prevalence of ESBL and KPC-producing *E. coli* among the hospitals

In this study, 76 patients were included, of which 33 were from male and female medicine wards, 15 from pediatric, 16 from neonatal and 12 from surgery wards. The median age of the patients in medicine, surgery, pediatric and neonatal wards was 35 (IQR: 28–45), 37 (IQR:33–50), 8 (IQR:7–10) years and 5 (IQR:4–7) days respectively. A total of 60 nasopharyngeal swabs were collected, and 7/60 (11.7%) were found to be positive for *E. coli*. Out of the 16 pus samples collected, 8/16 (50%) were positive for *E. coli*. With respect to patient hands, a total of 7/76 (9.2%) were positive for *E. coli*, whereas no *E. coli* was detected on the caregivers’ hands. In regards to the environmental samples, 16/76 (21.1%) of the bed pillows contained *E. coli*, followed by 8/76 (10.5%) of bed railings and 47/76 (61.8%) of floors (Table 1). In total, 523 *E. coli* isolates were obtained, which were subjected to testing for ESBL and KPC production.

**Table 1:** Percentage of samples positive for *E. coli* and ESBL *E. coli*.

	Hospital Name					
Sampling Point	Faridpur		Rajshahi		Rangpur	
	*E. coli* positive/Total (%)	ESBL *E. coli* positive/Total (%)	*E. coli* positive/Total (%)	ESBL *E. coli* positive/Total (%)	*E. coli* positive/Total (%)	ESBL *E. coli* positive/Total (%)
**Nasal Throat**	3/28 (10.7)	2/28 (7.1)	1/24 (4.2)	0/24 (0)	3/8 (37.5)	2/8 (25)
**Pus**	7/12 (58.3)	4/12 (33.3)	1/4 (25.0)	1/4 (25)	0/0 (0)	0/0 (0)
**Bed pillow**	8/40 (20)	5/40 (12.5)	6/28 (21.4)	3/28 (10.7)	2/8 (25)	1/8 (12.5)
**Bed Railing**	3/40 (7.5)	3/40 (7.5)	3/28 (10.7)	2/28 (7.1)	2/8 (25)	0/8 (0)
**Floor**	25/40 (62.5)	10/40 (25.0)	19/28 (67.9)	6/28 (21.4)	3/8 (37.5)	2/8 (25)
**Patient hand**	5/40 (12.5)	3/40 (7.5)	2/28 (7.14)	1/28 (3.6)	0/8 (0)	0/8 (0)
**Care giver hand**	0/40 (0)	0/40 (0)	0/28 (0)	0/28 (0)	0/8 (0)	0/8 (0)
Total	**51/240 (21.3)**	**27/240 (11.3)**	**32/168 (19)**	**13/168 (7.7)**	**10/48 (20.8)**	**5/48 (10.4)**

A total of 117/523 (22.4%) ESBL-producing *E. coli* were identified from various hospital environments and clinical samples. With regards to sampling locations, 4/60 (6.7%) nasopharyngeal swabs and 5/16 (31.3%) pus samples were positive for ESBL *E. coli*. Concerning patient hands, a total of 4/76 (5.26%) were positive for ESBL *E. coli*, with no ESBL *E. coli* present in caregivers' hands. For environmental samples, 9/76 (11.8%) of the bed pillows were positive for ESBL *E. coli*, followed by 5/76 (6.6%) of bed railings and 18/76 (23.7%) of floors (Table 1). A total of 109/117 (93.2%) of the ESBL *E. coli* were found to be KPC-producing. Among the ESBL isolates, 36/117 (30.8%) were from clinical and 81/117 (69.2%) were from environmental sources. From these isolates, 50/117 (42.7%) were isolated from swabs taken from the floor, 21/117 (18%) from the bed pillow, 7/117 (6%) from bed railing, 3/117 (2.6%) from patient's hands, 23/117 (19.7%) from nasal and throat samples of patients (nasopharyngeal), and 13/117 (11.1%) from wound pus samples. The distribution of ESBL and KPC-producing *E. coli* according to sampling location can be found in Table 2.

**Table 2:** Distribution of ESBL and KPC producing *E. coli* among different hospitals.

Hospital	Resistance	Bed Pillow	Bed Railing	Floor	Nasal Throat	Patients Hands	Pus Sample
		n (%)	n (%)	n (%)	n (%)	n (%)	n (%)
**Rajshahi**	ESBL-*E. coli*	16 (30.8)	2 (3.8)	26 (50.0)	3 (5.8)	–	5 (9.6)
	KPC-*E. coli*	15 (31.2)	2 (4.2)	23 (47.9)	3 (6.2)	–	5 (10.4)
**Faridpur**	ESBL-*E. coli*	4 (8.7)	–	18 (39.1)	14 (30.4)	3 (6.5)	7 (15.2)
	KPC-*E. coli*	4 (8.7)	–	18 (39.1)	14 (30.4)	3 (6.5)	7 (15.2)
**Rangpur**	ESBL-*E. coli*	1 (5.3)	5 (26.3)	6 (31.6)	6 (31.6)	–	1 (5.3)
	KPC-*E. coli*	1 (6.7)	1 (6.7)	6 (40.0)	6 (40.0)	–	1 (6.7)

During our study, all three hospitals harboured ESBL producing *E. coli*. The distribution of the isolates was rather diverse, with 46/178 (25.8%) isolates from Faridpur, 52/222 (23.4%) from Rajshahi and 19/123 (15.4%) from Rangpur being ESBL producing.

### 2.2 Significant number of ESBL isolates harboured resistance genes

**Fig. 1:**
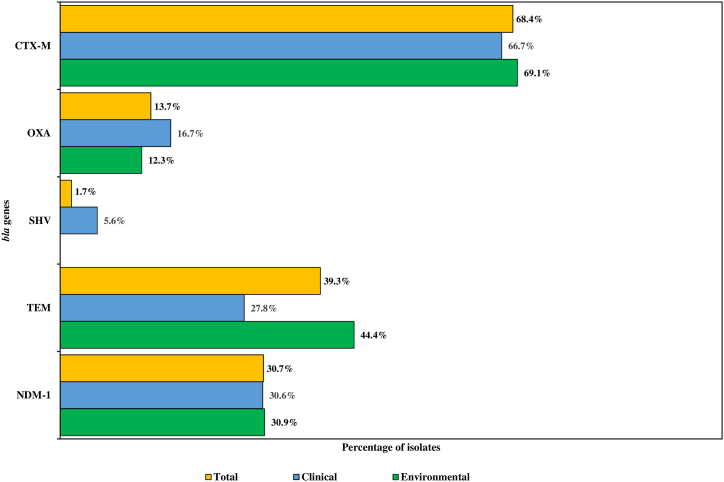
Distribution of β-lactamase genes among the ESBL *E. coli* isolates.

Among the ESBL *E. coli,* the presence of resistance genes was tested using PCR. Out of these, 79/117 (68.4%) were positive for *bla*_CTX-M_, 46/117 (39.3%) for *bla*_TEM_, 2/117 (1.7%) for *bla*_SHV_ and 16/117 (13.7%) isolates for *bla*_OXA_ (Fig. 1). Further, 36/117 (30.7%) of the isolates were positive for *bla*_NDM-1_ (Fig. 1)*.* Upon the branching of CTX-M groups, 74/79 (93.7%) of the isolates harboured *bla*_CTX-M-1_ group, 1/79 (1.3%) *bla*_CTX-M-9_ group, whereas none of the isolates were found to be positive for either *bla*_CTX-M-2_ or the *bla*_CTX-M-8_ group. Additionally, among the four genes tested, *bla*_TEM_ & *bla*_NDM-1_ coexisted in 29/117 (24.7%) of the isolates, *bla*_CTX-M_ and *bla*_NDM-1_ in 32/117 (27.35%), *bla*_TEM_ and *bla*_CTX-M_ in 33/117 (28.21%) and all three genes (*bla*_NDM-1_*, bla*_CTX-M_ *and bla*_TEM_) were found in 25/117 (21.37%) of the isolates.

For *bla*_NDM-1_ (environmental-30.9%, clinical-30.6%) and *bla*_CTX-M_ (environmental-69.1%, clinical-66.7%) genes, both environmental and clinical isolates were found to have similar rates of detection. However, for the *bla*_TEM_ (environmental-44.4%, clinical-27.8%) gene, higher detection rates were observed in environmental isolates, whereas *bla*_SHV_ (clinical-5.6%) was detected only in clinical isolates (Fig. 1).

### 2.3 Significant number of ESBL isolates harboured pathogenic genes

**Fig. 2:**
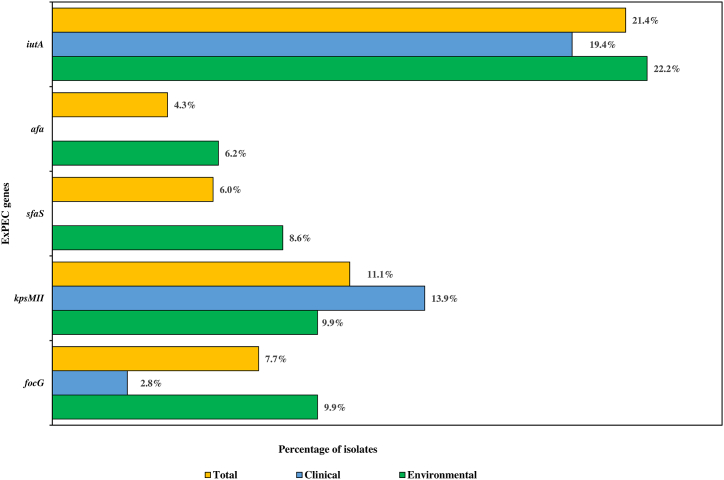
Distribution of virulence genes among the ESBL *E. coli* isolates.

Upon screening all the ESBL *E. coli* for diarrheagenic virulence factors, none of the isolates were found to harbour any of the tested genes. For the detection of extraintestinal pathogenic *E. coli*, the genes *focG, kpsMII, sfaS, afa* and *iutA* (five out of seven genes) were detected, and the distribution of the genes is depicted in Fig. 2. Out of the 117 ESBL *E. coli* tested, 42/117 (35.9%) isolates carried at least one ExPEC gene whereas none of the isolates carried all 7 tested genes. Among the environmental and clinical isolates, 9/117 (7.7%) isolates harboured *focG*, 13/117 (11.1%) *kpsMII*, 7/117 (6%) *sfaS*, 5/117 (4.3%) *afa*, 25/117 (21.4%) *iutA* and none of the isolates contained the *hlyD* or *papA* gene. The distribution of virulence genes among the environmental and clinical isolates exhibited significant diversity, as illustrated in Fig. 2. With respect to *iutA* (environmental-22.2%, clinical-19.4%) and *focG* (environmental-9.9%, clinical-2.8%) higher detection rates were observed among environmental isolates. In contrast, *kpsMII* (environmental-9.9%, clinical-13.9%) was detected at greater rates among clinical isolates, whereas, *sfaS* (environmental-8.6%) and *afa* (environmental-6.2%) were detected among environmental isolates only. Among the isolates harbouring more than one virulence factor, 4/117 (3.4%) isolates contained the gene *focG* and *kpsMII,* 6/117 (5.1%) *focG* and *sfaS,* 4/117 (5.1%) *focG* and *afa,* 3/117 (2.6%) *focG* and *iutA,* 3/117 *kpsMII* and *sfaS* (2.6%)*,* 2/117 (1.7%) *sfaS* and *afa*, 3/117 (2.6%) *sfaS* and *iutA,* 2/117 (1.7%) *focG, kpsMII, sfaS,* 2/117 (1.7%) *focG*, *sfaS, iutA,* and 2/117 (1.7%) *focG, kpsMII, sfaS and iutA.* If an isolate harbours 3 or more genes, it is termed an ExPEC strain [31]. A total of 6/117 (5.1%) isolates were found to be ExPEC as per criteria.

### 2.4 Presence of highly drug-resistant *E. coli* in healthcare facilities

**Fig. 3:**
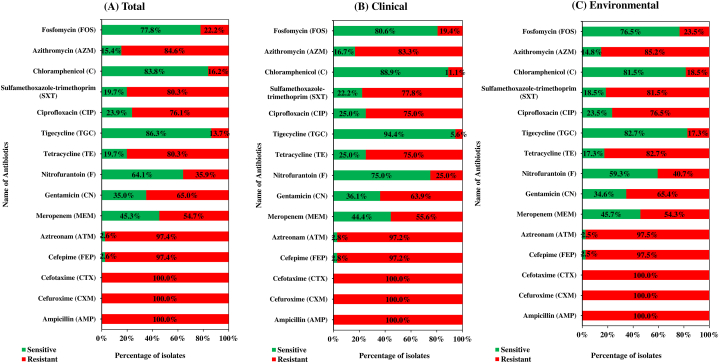
Antibiotic resistance patterns of the ESBL *E. coli* isolates. (A) Indicates resistance patterns among the total population of ESBL *E. coli* isolates, (B) indicates resistance patterns among clinical isolates and (C) indicates resistance patterns among environmental isolates.

Antibiotic susceptibility pattern was determined for 117 ESBL *E. coli*. All of the isolates were resistant to ampicillin, cefuroxime and cefotaxime. Notably, 114/117 (97.4%) were resistant to cefepime, 114/117 (97.4%) to aztreonam, 64/117 (54.7%) to meropenem and a large number of isolates exhibited varied resistance patterns to other antibiotics tested (Fig. 3). The most interesting finding was all the isolates were multidrug-resistant (MDR) with 30/117 (25.6%) of the isolates being highly drug-resistant (resistant to ≥ 12 antibiotic classes). The environmental isolates showed a higher percentage of resistance to fosfomycin (environmental-23.5%, clinical-19.4%), azithromycin (environmental-85.2%, clinical-83.3%), chloramphenicol (environmental-18.5%, clinical-11.1%), sulfamethaxazole-trimethoprim (environmental-81.5%, clinical-77.8%), ciprofloxacin (environmental-76.5%, clinical-75%), tigecycline (environmental-17.3%, clinical-5.6%), tetracycline (environmental-82.7%, clinical-75%), nitrofurantoin (environmental-40.7%, clinical-25%), gentamicin (environmental-65.4%, clinical-63.9%), aztreonam (environmental-97.5%, clinical-97.2%) and cefepime (environmental-97.5%, clinical-97.2%) (Fig. 3). Whereas, the clinical isolates showed a higher percentage of resistance to meropenem (environmental-54.3%, clinical-55.6%) (Fig. 3). The interpretation based on the diameter of zone of inhibition is given in Supplementary Table-S3.

### 2.5 ERIC fingerprinting differentiated the isolates into several clusters

The genetic relatedness of the ESBL *E. coli* was investigated using ERIC-PCR. The band patterns were calculated using the visibility and placements of gels according to molecular weights and molecular markers. Following dendrogram analysis (Supplementary Fig. S1), the isolates were separated into 12 clusters with a 70% similarity index. The isolates produced 4–15 amplicons, where 500bp and 2000 bp were common in most of the isolates. Furthermore, the largest cluster E−10 contained 43 isolates. The isolates (LEH-132 and LEH-130) obtained from the bed railing and nasal throat from the same hospital showed the highest similarity, suggesting that these isolates extend from the same clonal lineage (Supplementary Fig. S1). Likewise, LEH-60 and LEH-61 obtained from the floor of the same hospitals also showed similar genetic profiles. Most notably, LEH-26 isolated from the nasal-throat of a patient, was grouped with LEH-20 which was isolated from the corresponding floor to the patient of LEH-26. Likewise, the isolates LEH-110 isolated from the nasal throat and LEH-112 isolated from the corresponding floor were grouped under the same cluster. Interestingly, isolates from different sampling locations from the same hospital were found to be grouped under the same cluster.

### 2.6 Biofilm forming capability of ESBL producing *E. coli*

**Fig. 4:**
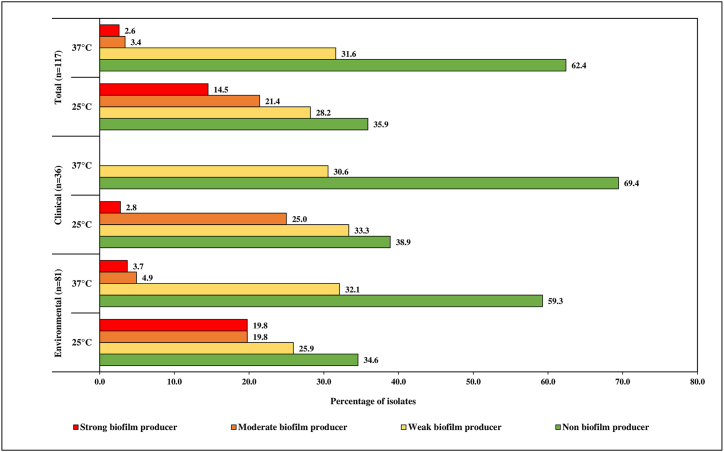
Biofilm producing capacity of the ESBL *E. coli* isolates.

A higher number of biofilm producers were observed at 25 °C, with a lower number of isolates exhibiting biofilm production at 37 °C. The number of isolates producing different categories of biofilm is depicted in Fig. 4. The degree of biofilm formation was observed with incubation temperatures of 25 °C and 37 °C. Regarding 25 °C, a total of 17/117 (14.5%) isolates were strong biofilm formers, 25/117 (21.4%) were moderate, 33/117 (28.2%) were weak and 42/117 (35.9%) were non-biofilm formers. In the case of 37 °C, a total of 3/117 (2.6%) isolates were strong, 4/117 (3.4%) were moderate, 37/117 (31.6%) were weak and 73/117 (62.4%) were non-biofilm formers. With respect to sampling locations, the environmental isolates formed a higher degree of biofilm at both 25 °C and 37 °C, in comparison to clinical isolates (Fig. 4).

### 2.7 Hierarchical clustering and correlation matrix revealed associations between clinical and environmental isolates

**Fig. 5:**
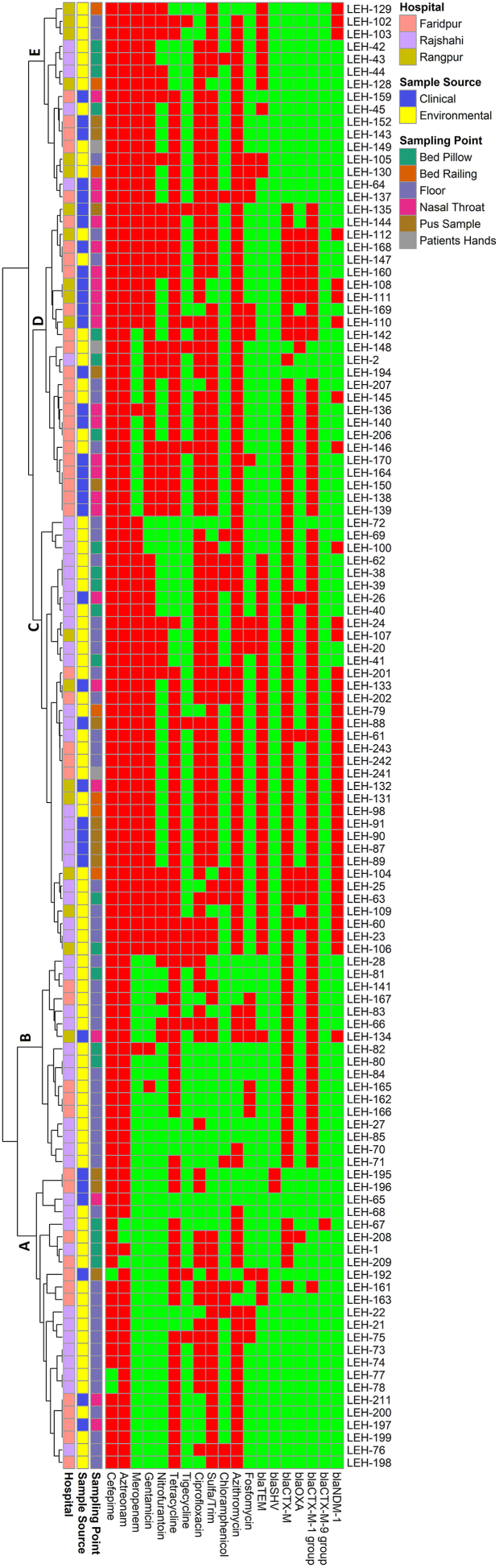
Heatmap and hierarchical clustering of *E. coli* isolates according to their phenotypic (antibiotic resistance) and genotypic (antibiotic resistance genes) profile of variables showing differences between isolates. Red color represented presence and green color represented the absence of resistance or gene. Left of the heatmap is a color representation of the different sources (patient in blue and environmental in red) and sampling points from those sources. Hierarchical clustering was performed using Wald's method and a binary distance matrix. Letters (A–E) designate the 5 main clusters described in the text.

**Fig. 6:**
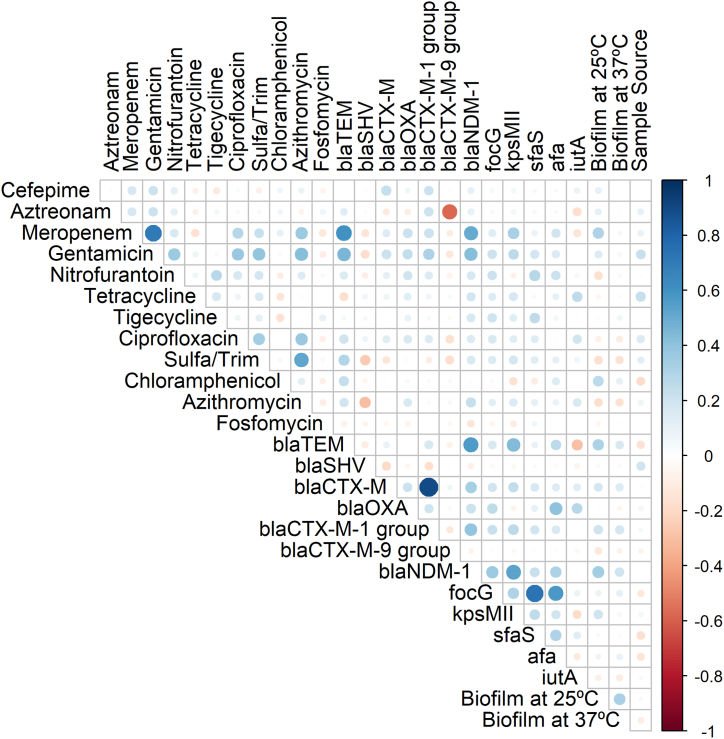
Correlation matrix of phenotypic (antibiotic resistance and biofilm formation ability) and genotypic (antibiotic resistance genes and virulence genes) features shows significant correlations. White spaces are not significantly correlated. Blue circles indicated a significant positive correlation and red circles show a significant negative correlation. The size and strength of colour represent the numerical value of the Phi correlation coefficient. The correlation matrix shows only significant (p < 0.05) associations, as assessed by the Chi-square test.

The associations between phenotypic and genotypic traits were explored using hierarchical clustering, alongside a heatmap (Fig. 5), and the use of a correlation matrix (Fig. 6). The results of the analysis showed a positive association between the presence of β-lactamase genes and phenotypic resistance to β-lactams. The resistance to carbapenems and aminoglycosides tested was significantly correlated with the presence of *bla*_TEM_ and *bla*_NDM-1_ genes (Fig. 6, p < 0.05).

Furthermore, the phenotypic resistance pattern indicated a co-occurrence of resistance for different antibiotics, and significant positive correlations were observed. For instance, resistance to azithromycin was positively correlated with meropenem, gentamicin, ciprofloxacin, and sulfamethoxazole-trimethoprim. Similarly, resistance to sulfamethoxazole-trimethoprim was positively associated with meropenem, gentamicin and ciprofloxacin (Fig. 6, p < 0.05). Additionally, the existence of virulence genes was correlated with the presence of resistance. In the case of *sfaS* and *afa,* both virulence genes showed positive correlations with resistance to nitrofurantoin and gentamicin. Similar co-existence of virulence genes and antibiotic resistance were observed and are depicted in Fig. 6. In the case of biofilm formation ability, significant correlations were observed between the presence of phenotypic and genotypic resistance, and the ability to form biofilm at 25 °C. In the case of 37 °C, similar positive correlations were observed but at reduced levels (Fig. 6, P < 0.05).

The segregation of 117 ESBL *E. coli* with respect to their phenotypic (AMR profiles) and genotypic (resistance gene presence) traits was obtained using hierarchical clustering. Interestingly, several isolates from both patient and environmental samples were grouped under the same cluster (Fig. 5). A total of five main clusters were identified. Cluster A consisted of 24 *E. coli* isolates, with six clinical and 18 environmental isolates. Cluster-C was the largest and consisted of 35 *E. coli* isolates, with eight from clinical, and 27 from environmental sources. Cluster-D was the second largest and consisted of 25 *E. coli* isolates, with nine environmental and 16 clinical isolates. Cluster-E was found to contain five clinical isolates and 11 enviromental isolates, with 16 *E. coli* isolates in total. Most notably, only one clinical isolate was grouped under Cluster-B, which consisted of 16 environmental isolates.

### 2.8 Genomic features

Table 3 and Supplementary Table-S1describe all the genomic characteristics of the sequenced isolates which include their GC content, length, coverage, coding sequences, N50, rMLST, phylotype, RNAs and the serotype of the isolates. The size of the genomes of the isolates was found to be ranging from 4.7 to 5.1 Mb, and their GC content ranged from 50.61% to 50.80%, with coverage from 86 to 167.

**Table 3:** Genomic characteristics of the ESBL *E. coli* isolates (Bioproject PRJNA904073).

ID	Isolates	Accession Number	Hospital	Sample Type	MLST^a^	FimH	Phylogroup	Serotype
LEH-23	RSNNBNT-13-FL	JARFNQ000000000	Rajshahi	Floor Swab	448	*fimH35*	B1	OUT:H8
LEH-24	RSMMBNT-14-FL-1	JARFNP000000000	Rajshahi	Floor Swab	973	*fimH95*	D	O11:H15
LEH-60	RSPDBNT-12-FL-1	JARFNO000000000	Rajshahi	Floor Swab	448	*fimH35*	B1	OUT:H8
LEH-147	FPMSBPS-12-FL-1	JARFNN000000000	Faridpur	Floor Swab	167	Unknown	A	O101: H9

a MLST-Multi-locus sequence typing.

### 2.9 In silico typing

The sequence diversity of the isolates based on the allelic combination of seven genes resulted in the four isolates belonging to 3 different sequence types (STs). The isolates LEH-23 and LEH-60 belonged to ST-448, LEH-24 belonged to ST-973, and LEH-147 belonged to ST-167. The serotyping of the isolates revealed that LEH-23 and LEH-60 belonged to the serotype OUT:H8, LEH-24 to O11:H15 and LEH-147 to O101:H9. The isolates LEH-23 and LEH-60 harboured *fimH35* allele, LEH-24 harboured *fimH95* allele, whereas no *fimH* allele could be identified in LEH-147. In silico typing predicted three different phylogroups for the isolates, with LEH-60 and LEH-23 belonging to phylogroup-B1, LEH- 24 to phylogroup-D and LEH-147 to phylogroup-A (Table 3).

### 2.10 Resistance gene profiling from WGS

A total of 26 genes conferring resistance to multiple antibiotic classes was identified. Among the genes conferring resistance to β-lactams and extended spectrum β-lactams, *bla*_TEM-1B_*, bla*_CMY-2_ and *bla*_NDM-5_ were present in LEH-23, *bla*_CTX-M-15_, *bla*_TEM-1B_ and *bla*_NDM-1_ in LEH-24 (Table 4). The genes *bla*_NDM-5_, *bla*_TEM-1B_, *bla*_CMY-2_, *bla*_CTX-M-15_ and *bla*_OXA-1_ were present in LEH-60, and the genes *bla*_CTX-M-15_, *bla*_OXA-1_, *bla*_CMY-146_ in LEH-147 (Table 4). The gene *dfrA12* involved in resistance to trimethoprim was present in LEH-23 and LEH-60, with *dfrA17* in LEH-60 and LEH-147. The gene *erm(B)* conferring resistance to macrolides was present in LEH-23, LEH-60 and LEH-147. The genes *catA1* and *catB3* involved in resistance to phenicol were detected in LEH-60. The gene *qnrS1* conferring resistance to quinolones found in LEH-24, along with the gene *tet(B)* in LEH-60 which mediates tetracycline resistance. The gene *sul1* involved in resistance to sulfonamides was detected in LEH-23, LEH-60 and LEH-147. Alongside, genes such as *mdf(A)* which confer resistance to multiple classes of antibiotics were also found to be present among all the sequenced isolates. The information related to gene presence can be found in Table 4.

**Table 4:** Overview of resistome and mobilome in ESBL *E. coli* isolates.

Isolates	Sample Type	β-Lactamase Resistance Genes	Fluoroquinolone Resistance Genes	Other Resistance Genes	Plasmids	pMLST
**LEH-23**	Floor	*bla*_NDM-5,_*bla*_TEM-1B_*, bla*_CMY-2_	*-*	*mdf(A), dfrA12, aadA2, sul1, rmtB, mph(A), erm(B)*	Col (BS512), IncFIB(AP001918), IncFII(29), IncFII(pAMA1167-NDM-5)	IncF [F44:A-:B49]
**LEH-24**	Floor	*bla*_TEM-1B_*,bla*_CTX-M-15_*, bla*_NDM-1_	*qnrS1*	*mdf(A), rmtB*	IncFII	IncF [F2:A-:B-]
**LEH-60**	Floor	*bla*_NDM-5,_*bla*_TEM-1B_*, bla*_CMY-2_*, bla*_CTX-M-15_*, bla*_OXA-1_	*aac(6′)-Ib-cr*	*mdf(A), dfrA12, aadA2, rmtB, mph(A), erm(B), aadA5, dfrA17, tet(B), sul1, catA1, catB3*	Col (BS512), IncFIA, IncFIB(AP001918), IncFII(29), IncFII(pAMA1167-NDM-5)	IncF [F48:A1:B49]
**LEH-147**	Floor	*bla*_CTX-M-15_*, bla*_OXA-1_*, bla*_CMY-146_	*aac(6′)-Ib-cr*	*mdf(A), sul1, mph(A), erm(B), aadA5, dfrA17, tet(B), aph(6)-Id, aph(3″)-Ib, sul2, aac(3)-Iia*	IncFIA, IncFIB(AP001918), IncFII, IncI(Gamma), IncY	IncF [F31:A4:B1]

### 2.11 Virulome profiling from WGS

Table 5 depicts the distribution of virulome among the *E. coli* isolates sequenced. The isolates displayed a high level of pathogenicity with a mean probability (P score) of 93.3%, with all isolates being human pathogens. The sequenced ExPEC isolates contained a total of 93 different virulence genes. The virulence factors (VFs) detected belonged to major functional categories including adhesins, protectins, invasins, iron uptake/siderophores and secretion systems among others (Table 5). The isolates LEH-23 and LEH-60 harboured 69 VFs (the highest number) followed by LEH-60 and LEH-147 harbouring 68 and 61 VFs respectively. In terms of virulence factors per category, LEH-23 and LEH-60 contained the highest number (25) of adherence factors, LEH-147 contained the highest number (29) of iron uptake factors, LEH-23 and LEH-60 had the highest number (4) of protectins and invasins. Whereas, all the sequenced isolates had an equal number (11) of secretion system factors.

**Table 5:** In silico identification of human pathogenicity and virulence factors in the ESBL-*E. coli* isolates.

Isolate Name	LEH-23	LEH-24	LEH-60	LEH-147
**Pathogenicity Score (No. of pathogenic families)**	0.936 (717)	0.936 (400)	0.935 (732)	0.924 (703)
**Human Pathogenicity**	Yes	Yes	Yes	Yes
**Virulence Factors**				
**Adherence**	*afaB-I, afaC-I, csgB, csgD, csgF, csgG, daaF, fdeC, fimA, fimB, fimC, fimD, fimE, fimF, fimG, fimH, fimI, ompA, papX, ecpE, ecpD, ecpC, ecpB, ecpA, ecpR*	*csgB, csgD, csgF, csgG, fdeC, fimA, fimB, fimC, fimD, fimE, fimF, fimG, fimH, fimI, ompA, ecpE, ecpD, ecpC, ecpB, ecpA, ecpR*	*afaB-I, afaC-I,csgB, csgD, csgF, csgG, daaF, fdeC, fimA, fimB, fimC, fimD, fimE, fimF, fimG, fimH, fimI, ompA, papX, ecpE, ecpD, ecpC, ecpB, ecpA, ecpR*	*csgB, csgD, csgF, csgG, fdeC, ompA, ecpE, ecpD, ecpC, ecpB, ecpA, ecpR*
**Iron Uptake**	*entA, entB, entC, entD, entE, entF, entS, fepA, fepC, fepG, fepD, fepB, fes, fyuA, irp1, irp2, ybtS, ybtX, ybtQ, ybtP, ybtA, ybtU, ybtT, ybtE*	*entA, entB, entC, entD, entE, entF, entS, fepA, fepC, fepG, fepD, fepB, fes,*	*entA, entB, entC, entD, entE, entF, entS, fepA, fepC, fepG, fepD, fepB, fes, fyuA, irp1, irp2, ybtS, ybtX, ybtQ, ybtP, ybtA, ybtU, ybtT, ybtE*	*entA, entB, entC, entD, entE, entF, entS, fepA, fepC, fepG, fepD, fepB, fes, fyuA, irp1, irp2, iucA, iutA, iucD, iucC, iucB, ybtS, ybtX, ybtQ, ybtP, ybtA, ybtU, ybtT, ybtE*
**Secretion System**	*gspM, gspL, gspK, gspJ, gspI, gspH, gspG, gspF, gspE, gspD, gspC*	*gspM, gspL, gspK, gspJ, gspI, gspH, gspG, gspF, gspE, gspD, gspC*	*gspM, gspL, gspK, gspJ, gspI, gspH, gspG, gspF, gspE, gspD, gspC*	*gspM, gspL, gspK, gspJ, gspI, gspH, gspG, gspF, gspE, gspD, gspC*
**Protectins and Invasins**	*draA, draD, draP, draE2*	*kpsD, kpsM*	*draA, draD, draP, draE2*	*-*
**Miscellaneous**	*espL1, espR1, espX1, espX4, espX5*	*aslA, chuA, chuS, chuT, chuW, chuY, chuU, chuV, espL1, espL4, espR1, espR3, espX1, espX4, espX5 espX2, espY1, espY2, espY4, espY3, shuX*	*espL1, espR1,espX1, espX4, espX5*	*aslA, espL1, espL4, espR1, espR4, espX1, espX4, espX5, espY1*

### 2.12 Mobile genetic elements

The analysis of the WGS data revealed eight different plasmid replicons among the sequenced isolates, with the isolates harbouring multiple replicon types concomitantly. In regards to plasmid replicons, seven different incompatibility (Inc) plasmid replicons were identified, including IncFIB (AP001918), IncFII (29), IncFII (pAMA1167-NDM-5), IncFII, IncFIA, IncI (Gamma) and IncY**.** All isolates harboured the IncF types with a single isolate (LEH-147) harbouring the IncY and IncI types. The in-silico plasmid MLST analysis designated STs to the plasmid incompatibility groups, with IncF being assigned the ST F44:A-:B49, F2:A-:B- and F31:A4:B1. The highest number of insertion sequences was observed for LEH-23 (13), followed by LEH-60 (12), LEH-147 (12) and LEH-24 (4). In contrast, both LEH-147 and LEH-23 harboured 7 CRISPR arrays, with LEH-24 and LEH-60 containing 1 and 4 CRISPR arrays respectively. The highest number of tandem repeats was found in LEH-147 (96), followed by LEH-24 (27), LEH-23 (18) and LEH-60 (18). Additionally, all the isolates harboured prophage sequences, with the highest number being observed in LEH-147 (Table 6).

**Table 6:** Mobile genetic elements detected in the ESBL *E. coli* isolates.

Isolates	Plasmid Inc groups	Insertion Sequence	Phages	CRISPR Array (Cas System)	Tandem Repeats
**LEH-23**	Col (BS512), IncFIB(AP001918), IncFII(29), IncFII(pAMA1167-NDM-5)	IS903,MITEEc1,IS5, ISKpn8, ISEc1, IS5, IS911, ISEc30, IS6100, ISEc38, ISEc9, IS100, IS26	PHAGE_Klebsi_4LV2017_NC_047818, PHAGE_Entero_mEp460_NC_019716	7 (Cas3)	18
**LEH-24**	IncFII	MITEEc1, ISEc46, ISEc9, IS26	PHAGE_Entero_mEp460_NC_019716	1(Cas3)	27
**LEH-60**	Col (BS512), IncFIA, IncFIB(AP001918), IncFII(29), IncFII(pAMA1167-NDM-5)	IS903, MITEEc1, ISEc30, ISKpn8, IS5, ISEc1, IS911, IS6100, ISEc38, ISKpn24, IS100, IS26	PHAGE_Klebsi_4LV2017_NC_047818, PHAGE_Entero_cdtI_NC_009514	4(Cas1,Cas3,Cas2,Cas6,Csy1,Csy2,Csy3)	18
**LEH-148**	IncFIA, IncFIB(AP001918), IncFII, IncI(Gamma), IncY	ISEc1, MITEEc1, IS609, ISEc78, ISEc38, IS5075, ISSfl10, ISKpn8, IS421, ISKox3, IS3, IS102	PHAGE_Entero_mEp460_NC_019716, PHAGE_Salmon_SJ46_NC_031129, PHAGE_Escher_pro483_NC_028943, PHAGE_Entero_I2_2_NC_001332	7 (Cas1,Cas2,Cas3,Cas5,Cas6,Cas7,Cse1,Cse2)	96

### 2.13 Phylogenetic analysis

**Fig. 7:**
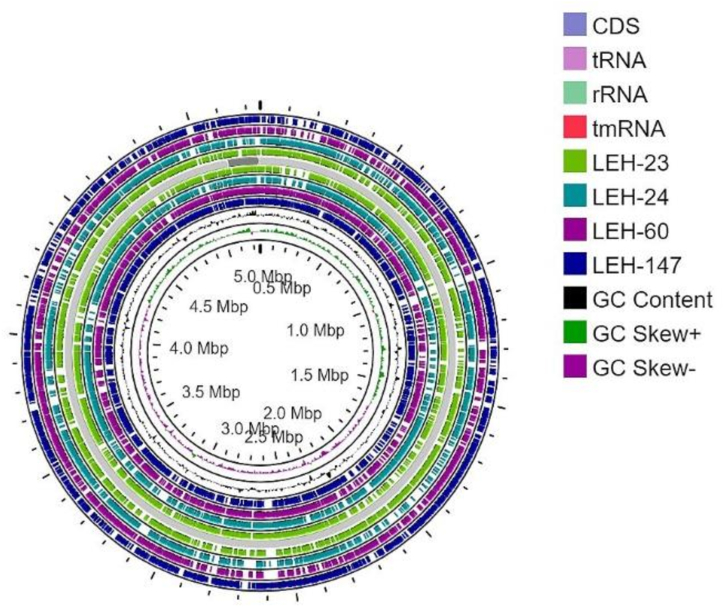
Circular genome representation of selected ESBL-producing *E. coli* aligned with reference genome. Circular map of selected ESBL-producing *E. coli* (LEH-23, LEH-24, LEH-60, LEH-147) with comparative alignment against *E. coli* EC-958.v1 (GCA_000285655.3), generated using Proksee Server.

**Fig. 8:**
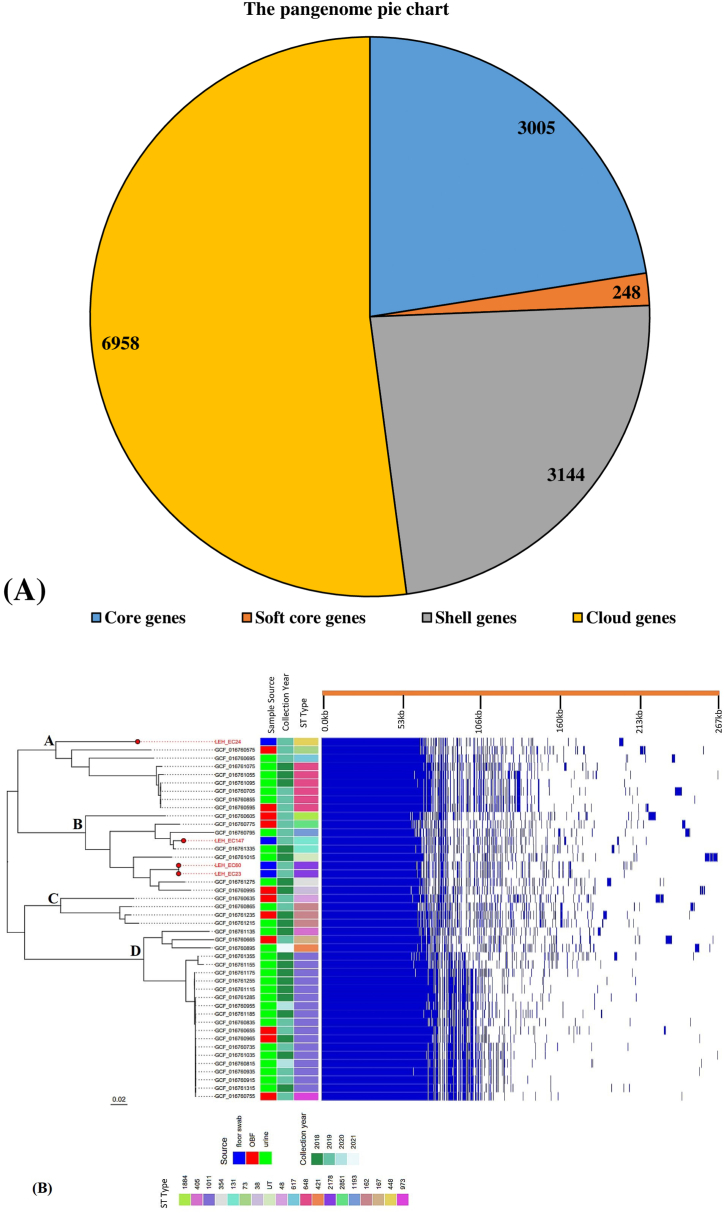
**(A)** A pangenome pie chart presentation of LEH-23, LEH-24, LEH-60, LEH-147 and 46 other strains of Bangladeshi origin. The pie chart shows the proportion of core and accessory genes present in the strains. **(B)** Maximum likelihood phylogenetic tree based on the core genomes. Four isolates from our study were highlighted with red tips. Metadata of the selected isolates including sample source, and year of collection presented in the middle. The right side represents the matrix of the accessory and core genes of the isolates by blue (present) and white (absent) fragments.

The contigs of the sequenced isolates were analyzed and plotted against the genome of EC-958.v1 (GCA_000285655.3) for visualizing their genomic organization. The comparative analysis revealed identical and distinctive regions of the sequenced isolates toward the reference genome (Fig. 7). The pangenome of 46 Bangladeshi strains and four sequenced strains consisted of 13355 genes, with 3005 core genes present in 99–100% of the strains. A total of 248 soft core genes were present in 95–99% of the strains, 3144 shell genes in 15–95%, and 6958 cloud genes in 0–15% of the strains (Fig. 8A). Accessory genes were considered to examine the similarities among strains. In phylogenetic analysis, all strains are divided into four major clusters. Three sequenced strains LEH-23, LEH-60, and LEH-147 were distributed in cluster B. Interestingly, LEH-24 is distributed in a unique clade in the phylogenetic tree (Fig. 8B).

### 2.14 Nucleotide accession number

The sequences of the ESBL *E. coli* isolates have been deposited at NCBI, under the bioproject accession number PRJNA904073. *E. coli* strains LEH-23, LEH-24, LEH-60 and LEH-147 are available under the accession number JARFNQ000000000, JARFNP000000000, JARFNO000000000 and JARFNN000000000 respectively.

## 3 Discussion

The alarming increase in the rates of antimicrobial resistance is a global cause for concern, and the World Health Organization (WHO) has listed ESBL producing Enterobacteriaceae on its critical priority list requiring the formulation of new treatments [32]. Bangladesh, alongside other South-Asian countries, is contained within the WHO regions which are burdened with the highest risk of AMR [33]. Several strategies to combat AMR rely on a periodic surveillance system, assessment of threats and an improvement in healthcare policies developed from implementing the findings. Therefore, a scarcity of data relating to the scenario of AMR, the presence of antibiotic resistance genes (ARGs), molecular determinants & whole genome sequence analysis of *E. coli* existing within hospital environments prompted this study. This present study aimed to investigate the phenotypic and molecular characterization of ESBL *E. coli,* testing for the presence of resistance and virulence genes, biofilm formation capability, determination of antibiotic susceptibility profiles and whole genome sequencing of the pathogenic highly drug resistant isolates.

In our study, 523 *E. coli* were isolated from various environmental and clinical specimens, among which 117 (26%) isolates were positive for ESBL production. In comparison to studies worldwide with rates ranging from 44 to 91%, the detection rates observed in this study are proportionately lower [[34], [35], [36]]. The difference in the detection rates may be due to changes in sampling locations, variations in techniques used, or primarily caused due to a lower prevalence of ESBL *E. coli* in tested environments. Among the environmental isolates, a large portion has been detected from the floors (42.7%), bed pillows (18%), patient hands (2.6%) and bed railings (6%). Although to our knowledge, this is the first study assessing the presence of ESBL *E. coli* among healthcare environments in Bangladesh; studies conducted previously support our findings, as similar isolates have been well known to colonize inanimate hospital surfaces [35]. High rates of bacterial prevalence in hospital environments are a well established route for contracting hospital acquired infections (HAIs) [37]. Overcrowding, lack of adequate beds combined with patients placed on hospital floors and hallways, and inadequate sanitary practices among the facilities, all present an increased risk of resistant bacterial transmission and contraction of HAIs among the patients [38].

Additionally, among the clinical isolates, a significant portion of the isolates were from nasal throats (19.7%), and pus samples (11.1%). In Bangladesh, higher rates (26.5%–33.9%) of ESBL *E. coli*, have been recorded among clinical samples, and our findings are comparable to those previously detected [24]. The difference between the rates detected previously and those observed in this study may be attributed to selection bias, variance in the number of isolates, sampling locations and use of phenotypic confirmation. These findings suggest a widespread prevalence of ESBL *E. coli* in hospital patients of Bangladesh, and direct patient-caregiver contact, proximity droplet formation and contact with healthcare workers are all risk factors that might play a role in disseminating HAIs [39].

Among the 117 ESBL producers in our study, a large number of isolates were positive for β-lactamase having clinical significance. The primary groups of ESBL genes prevalent among nosocomial bacteria are *bla*_CTX-M_*, bla*_SHV_ and *bla*_TEM_ [40]. Further, previous studies have shown the prevalence of *bla*_OXA_ among isolates of *E. coli*, and the rates detected in this study suggest an overall decrease in prevalence [41]. The difference in rates may be due to a high percentage of environmental isolates included in the study. Similar to previous studies, *bla*_CTX-M_ was the most prevalent gene among the isolates [24,40]. Several clones of the CTX-M enzyme have been reported worldwide, and it has been shown that the spread of these clones may follow an epidemic pattern aided by the dissemination of multiple specific clones or mobile genetic elements [7]. This study found that several ESBL *E. coli* contained multiple CTX-M types, with CTX-*M*-1 group being present in 93.7% (74/79) of the isolates and CTX-*M*-9 group being present in 1.3% (1/79). Previous reports have identified a high prevalence of CTX-*M*-1 group (52%–59%) similar to our study and is indicated to be the most common CTX-M group among *E. coli* isolates [25,36]. Several studies indicate that the increase of CTX-Ms in hospital settings is a consequence of these enzymes being introduced from the community as opposed to emerging and expanding from nosocomial settings [42]. The high prevalence of CTX-Ms uncovered in this study reinstates the importance of finding an efficacious solution.

The present study revealed the presence of *bla*_NDM-1_ in ESBL *E. coli* among hospital environments and patient samples. A total of 36/117 (30.7%) NDM-1 positive *E. coli* were found. Since its first identification in 2008, NDM-1 has grasped worldwide attention due to its rapid spread among Enterobacteriaceae in clinical isolates and contaminating environments [43]. A high prevalence of NDM has been reported in the Indian subcontinent and the Middle East [43]. In Bangladesh, NDM-1 was initially found in *K. pneumoniae* and since then has been discovered in *E. coli* [44,45]. Additionally, NDM-producing *E. coli* has been found in wastewater samples of adjacent hospital areas, and the clinical detection of the gene has also been observed [45,46]. The rates detected in this study suggest an overall increase in the prevalence of NDM-1 among *E. coli* isolates, with previous detection rates of 3.5–29% being observed in Bangladesh [24,45,47]. The increase in the prevalence of NDM-1 among *E. coli* isolates may be linked to the increased use of carbapenems in Bangladesh [24].

*E. coli* are well-known biofilm formers on biotic and abiotic surfaces, such as catheters and endotracheal tubing [48]. Among the tested isolates, both clinical and environmental, exhibited various degrees of biofilm formation at 25 °C and 37 °C. In particular, it was found that clinical isolates were more likely to form weak to moderate biofilms. However, previously it has been observed that clinical samples are likely to form a higher degree of biofilms [49]. The biofilm formation capacity of *E. coli* may differ between isolates due to several factors affecting growth such as physicochemical parameters, physical interaction between the media, type of surface for attachment, temperature and pH [50]. The number of biofilm forming isolates at 25 °C was higher, which is in agreement with a previous study showing a greater number of biofilm formers at similar temperatures [51].

The extensive use of antimicrobials in healthcare settings and medical treatment has led to a high incidence of AMR *E. coli* [52]. Multidrug-resistant ESBL *E. coli* is a primary concern due to their role in nosocomial infections which are not easily treatable, particularly in countries where access to antibiotics may be difficult [53]. In our study, all ESBL *E. coli* were MDR and 30 out of 117 isolates were classified as highly drug resistant. This increased ratio of resistance to multiple antibiotic classes among ESBL *E. coli* may indicate the selection of co-resistance. There is a lack of information available on such high incidences of MDR *E. coli* in hospital environments and clinical samples in Bangladesh. However, our results are comparable to studies previously conducted worldwide [52]. The excessive use of antibiotics, transfer of resistance genes, and gradual accumulation of mutations are all factors that may explain the high prevalence of antibiotic resistance [54]. Among the isolates in this study, all were resistant to ampicillin, cefuroxime and cefotaxime. The resistance observed to these antibiotics is due to the selection of ESBL producing *E. coli* which are resistant to β-lactams [8]. Notably, a large percentage of isolates were resistant to cefepime (97.4%), which is a fourth generation cephalosporin. Additionally, a high percentage of ESBL *E. coli* were resistant to quinolones (76.1%), compared to the median (65.2%) rates around the country, indicating an increase in resistance [55]. The increased resistance may result from excessive use of these antibiotics frequently sold and used within the country [56].

Further, high rates of resistance against monobactams (aztreonam), aminoglycosides (gentamicin), nitrofurans (nitrofurantoin), sulfonamides (sulfamethoxazole-trimethoprim) and macrolides were observed, which are in accordance with previous studies [52]. Our findings suggest that multidrug-resistant *E. coli* are widely disseminated within hospital environments and their patients, including extended spectrum beta-lactam resistant isolates which are a significant hindrance for physicians who face a scarcity of therapeutic options available for treatment [52].

ExPEC strains of bacteria are often linked with bloodstream, urinary tract and prostate infections at sites that are non-intestinal. They are part of the normal flora of the intestine and form a reservoir, from which extraintestinal infections are caused [57]. Among the isolates tested, at least one virulence gene was found in 35.9% (42/117) whereas 5.1% (6/117) were detected to be ExPEC strains. ExPEC strains of bacteria have been well known to colonize hospital environments and patients, and previous studies have reported the isolation of similar strains [58]. Additionally, all the isolates were screened for diarrheagenic genes. However, none of the isolates were found to contain any of the related genes. The diarrheagenic genes tested are usually found in gastrointestinal infection causing *E. coli* [13]. Therefore, *E. coli* that are responsible for causing extraintestinal infections may contain different virulence factors, owing to their transmission routes and infection causing abilities [59].

Genetic fingerprinting patterns have been obtained for 117 ESBL *E. coli* isolates using ERIC-PCR. The dendrogram construction resulted in 12 clusters at a 70% similarity index. The largest cluster was E−10 with 43 isolates, demonstrating the genetic linkage among them although they may differ in their phenotypic and genotypic characteristics. These findings were similar to a previous study that reported high genetic diversity of *E. coli* in the environmental and clinical samples [13,60]. The primary reason for this genetic variation may be the bacterial genome change due to host and environmental persistence [61] The isolates screened during this study exhibited a wide range of genetic variations. The major implication of this result is that ERIC-PCR analysis can be used as an effective method for routine surveillance of healthcare facilities for the clonal linkage of ESBL *E. coli*.

The coexistence of virulence and antibiotic resistance is of particular concern, and this study revealed a significant positive correlation between the presence of virulence genes and multidrug-resistance. These findings are in agreement with previous studies from Bangladesh [13,25]. Regarding hierarchical clustering, the isolates from both hospital environments and clinical samples were grouped under the same clusters, expressing the similarity of the isolates and their potential ability to spread from the environment onto hospital patients.

In the present study, the genomes of four highly drug resistant pathogenic isolates designated LEH-23, LEH-24, LEH-60 and LEH-147 were explored using WGS to understand the molecular mechanisms of resistance. The sequenced isolates showed the presence of multiple resistant genes which exhibited resistance against β-lactams, fluoroquinolones, tetracyclines and sulphonamides (Table 4). These findings are further supported by the resistant phenotype and genotype of the isolates resulting from antibiotic susceptibility testing and PCR screening. MLST was used to determine the epidemiological relatedness of the isolates; LEH-23 and LEH-60 belonged to the ST-448, whereas LEH-24 and LEH-147 belonged to the ST-973 and ST-167 respectively. The ST-448 has been known to exhibit multidrug-resistance and was previously isolated from environmental and clinical isolates [62]. Further, the ST-167 and ST-973 have been known to be pathogenic and were previously isolated from clinical samples [46,63], whereas the ST-167 has been associated with multiple outbreaks in the US [64]. LEH-23 and LEH-60 belonged to the phylogroup B1, the predominant phylotype in uropathogenic strains known for sepsis [65], and associated with extra-intestinal infections, which is expected as the sequenced isolates are ExPEC strains [66]. The isolates LEH-24 and LEH-147 belonged to the phylogroup D and A respectively, with both groups being associated with urinary tract infections [67].

The isolates sequenced were identified as human pathogens with an average 93.3% score (Table 5). The presence of a large number of VFs and ARGs may be the cause of such extreme pathogenicity [46]. These findings suggest that environmental bacteria in healthcare facilities are reservoirs of virulence and resistance genes, which may be passed onto non-pathogenic bacteria through horizontal gene transfer. As a result, these new strains of bacteria may transmit to humans and patients through contact with inanimate objects and this finding indicates the need for a reemphasis of infection control and adequate preventive measures in healthcare facilities.

The study of mobile genetic elements helps in understanding the spread and mobility of resistance genes and virulence factors between bacterial species, and pathogenic and non-pathogenic strains. The sequenced isolates were found to harbour multiple plasmids belonging to the major replicon types, and IncF was the most common (Table 6). Several studies have shown that IncF plasmid is known to carry ARGs in *E. coli*, which may be the possible reason behind the highly drug resistant properties of the sequenced isolates [68]. The widespread presence of ESBL *E. coli* in patients and hospital environments suggests that these plasmids may likely cause long-term resistance and pathogenicity in the environmental microbiome.

The current study has a few limitations that should be acknowledged and considered. First, due to budget and time restrictions, all types of clinical samples and needed epidemiological data could not be included, limiting the broader relevance of our findings. Second, the lack of conjugation studies limited our capacity to investigate the transferability of mobile genetic components, potentially hindering our understanding of the role of horizontal gene transfer in the spread of antibiotic resistance. Third, while PCR amplification revealed helpful information, the lack of sequencing data for the amplicons limited our ability to identify genetic variations driving resistance to ESBLs. Finally, our study did not investigate the migration and dissemination of ESBL *E. coli* between environmental and clinical isolates, nor did it investigate the transmission of resistance genes between these contexts, leaving critical questions about bidirectional resistance exchange unanswered. Despite these limitations, our findings provide important insights into ESBL prevalence and resistance genes, laying the foundation for future studies to fill gaps in our understanding of antibiotic resistance dynamics.

To conclude, our findings indicate a high prevalence of ESBL *E. coli* within the hospital environments, with most isolates being resistant to frequently used antibiotics. Additionally, the isolates possess a high capacity to form biofilms and genetic determinants associated with virulence and antibiotic resistance. These findings reveal the overall scenario of contamination within hospital environments, for which quick actions are required to prevent resistance development and to combat the incidence of hospital acquired infections. A change in healthcare policies that prioritize optimum treatment, rigorous infection prevention and control, and adequate sanitary practices should be implemented. Furthermore, future research should be conducted to establish a standardized ‘One Health’ approach for surveillance of ESBL *E. coli* and other Enterobacteriaceae in hospital environments, which may generate crucial data required for intervention and policy change.

## 4 Methods

### 4.1 Sampling

**Fig. 9:**
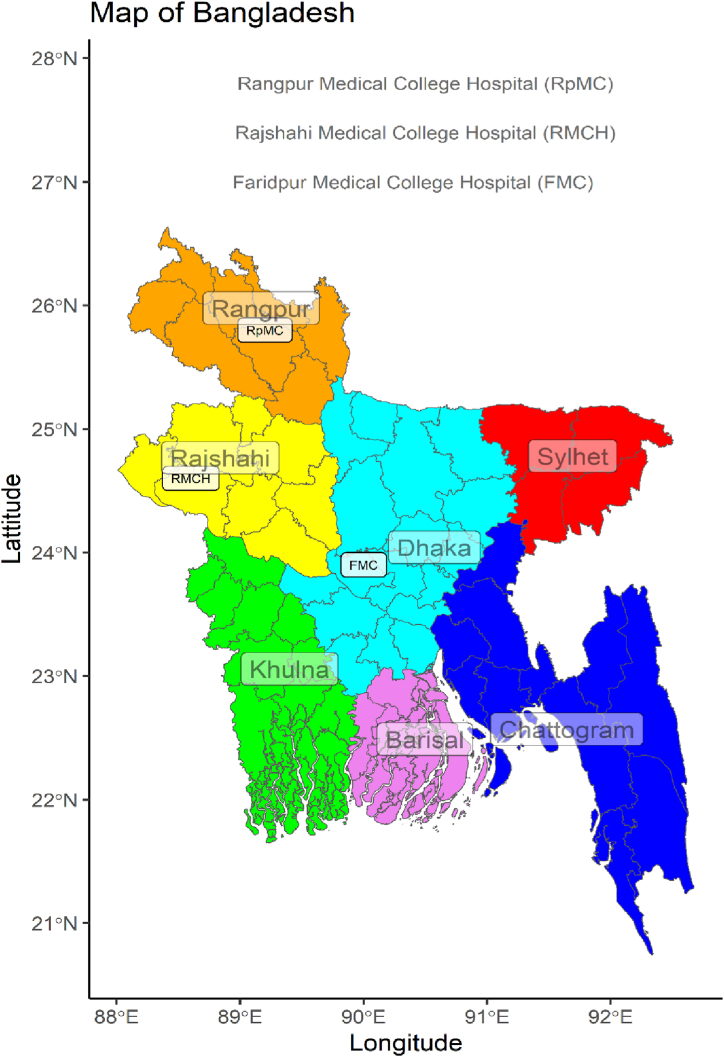
Geographical locations of the studied hospitals. Each hospital has several wards, including adult male and female medicine, surgery, paediatrics, and neonatal wards.

The samples were collected from Faridpur, Rajshahi and Rangpur Medical College Hospital (Fig. 9) with prior approval of the Institutional Review Board (IRB) of the International Centre for Diarrhoeal Disease Research, Bangladesh (icddr,b). The study included multiple wards within these hospitals including male and female medicine, pediatrics, surgery and neonatal. Clinical samples were collected from patients, including nasal-throat samples, pus samples, and samples from both patient hands and caregivers’ hands. A total of 76 patients were included in the study, with 16 of them were suffering from an active infection, while the remaining 60 patients showed no signs of active infection. Additionally, samples were collected from corresponding surfaces (area of 100 cm^2^) of bed pillows, bed railings and floors. These surfaces were wiped with a cotton swab (Puritan, Maine, USA) moistened with sterile physiological saline and stored in 15 ml conical tubes (Corning, NY, USA) containing 10 ml phosphate buffered saline (PBS). After collection, the samples were transported to the Laboratory of Environmental Health, icddr,b, Dhaka maintaining the cold chain according to the standard procedures [69].

### 4.2 Sample processing and isolation of ESBL and KPC positive *E. coli*

For analysis, the samples were brought to room temperature, and then they were thoroughly vortexed to ensure a uniform mixture. Following homogenization, the samples were prepared, and 1000 μl and 100 μl of each sample were filtered with the addition of 10 ml of autoclaved PBS through a 0.22 μm membrane filter (Sartorius Stedim, Goettingen, Germany). The membrane filters were then placed on mTEC (Modified Thermotolerant *E. coli*) agar plates (BD Difco, NJ, USA). The plates were then incubated at 37 ± 0.5 °C for 2 h followed by further incubation for 22–24 h at 44 ± 0.5 °C. After incubation, colonies displaying a magenta color were considered putative *E. coli*. For the subsequent screening and isolation of ESBL and *Klebsiella pneumoniae* carbapenemase (KPC) producing *E. coli*, the patch inoculation method was employed, following a previously published protocol [13]. Additionally, positive controls were used for both ESBL and KPC media, ESBL *E. coli* CIP 103982 and *E. coli* IMP NCTC 13476 were used as positive controls respectively, as recommended by the manufacturer. *E. coli* ATCC 25922 was used as negative control.

### 4.3 Detection of antibiotic resistance genes

The DNA from the samples was isolated using the boiling lysis method [13]. Subsequently, the DNA content was then subjected to PCR analysis to determine the presence of antibiotic resistance genes, *bla*_SHV_*, bla*_TEM_*, bla*_CTX-M_ and *bla*_OXA_ according to the previously published protocol [70]. Positive controls for the reactions were obtained from previous studies [13]. Furthermore, the isolates were examined for the presence of the following genes: *bla*_NDM-1_*, bla*_CTX-M-1_ group, *bla*_CTX-M-9_ group*, bla*_CTX-M-2_ group and *bla*_CTX-M-8_ group by using PCR methods described in a previous study [13]. Likewise, isolates obtained from the previous study were utilized as positive controls. The primer sequences and corresponding product sizes are listed in Supplementary Table-S2. To detect the presence and subsequent amplification of the genes being examined, the PCR products were resolved in 1% agarose gel with 0.5X tris borate EDTA buffer. The gels were then observed, and images were taken on GelDoc Go Imaging System (BIORAD, California, USA).

### 4.4 Detection of pathogenic *E. coli*

The ESBL producing *E. coli* were subjected to PCR analysis to determine the presence of diarrheagenic genes, following a previously published protocol [13]. Additionally, the isolates were screened for the presence of seven extraintestinal pathogenic *E. coli* (ExPEC) associated genes. Two separate multiplex PCRs were carried out to identify ExPEC associated genes, according to the previously published protocol [13]. The primer sequences, corresponding product sizes and associated pathotypes are listed in Supplementary Table-S2.

### 4.5 Determination of antibiotic resistance profiles

The antibiotic susceptibility profiles of all the isolates were determined following the guidelines provided by the Clinical Laboratory Standards Institute (CLSI) [71]. The recommended Kirby-Bauer disk diffusion method was utilized for susceptibility testing. The isolates were screened for susceptibility against 15 different antibiotics from 14 different classes. Commercially available disks (Thermo Fisher Scientific™, Waltham, USA) were used for this purpose. The list of antibiotics used, along with their concentrations can be found in Supplementary Table-S3.

### 4.6 Molecular typing of ESBL *E. coli*

To determine the genetic similarity of the isolates, the Enterobacterial Repetitive Intergenic Consensus (ERIC) sequences was used. The PCR was conducted following a previously published protocol [72], utilizing the primer listed in Supplementary Table-S2. For gel image analysis, GelJ v.2.0 [73] was used, which employs the Gaussian regression for image normalization. The ERIC-PCR patterns were clustered and a phylogenetic tree was generated using the Unweighted Pair Group Method with Arithmetic Mean (UPGMA) with a 1.0% tolerance value. The UPGMA algorithm calculates genetic distances between isolates and constructs a hierarchical tree based on these distances.

### 4.7 Biofilm formation assay

The biofilm formation capability of the isolates was assessed using the quantitative adherence assay [74]. After the biofilm formation, the optical density (OD) of the microtiter plate was measured at a wavelength of 590 nm using an ELISA plate reader (BioTek, Vermont, USA). Based on a previously published protocol, the isolates were categorized either as strong, moderate, weak or non-biofilm formers, depending on the biofilm formation capacity [75].

### 4.8 Statistical analysis

In the statistical analysis, the antibiotic resistance gene summaries, as well as the presence or absence of resistance and virulence genes, were converted to binary coding using 1 and 0. A value of 1 represented the presence of a given gene (for example *bla*_NDM-1_) while a value of 0 indicated its absence. The statistical programming language R (V: 4.1.1) was utilized to conduct the analysis [76]. The ‘corr’ function was used to generate correlations among the variables and the ‘corr.test’ function was employed to determine the significance (p < 0.05) using Pearson correlation. The ‘corrplot’ function was used to visualize the significant correlations between the variables [77]. To visualize clusters of the samples and features, hierarchical clustering was performed using the ‘pheatmap’ function from the “pheatmap” package. Ward's approach was employed for distance matrices [78]. It is worth mentioning that the correlation plot excluded the variables with a hundred percent resistance rate to ampicillin, cefotaxime, and cefuroxime, as their correlation did not demonstrate any significance in depicting the correlation plot.

### 4.9 Whole genome sequencing of highly drug resistant pathogenic isolates

Four isolates, namely LEH-23, LEH-24, LEH-60, and LEH-147, were selected for whole-genome sequencing (WGS) based on their high drug resistance and pathogenicity. These isolates were obtained from environmental sources. Genomic DNA was extracted and purified from an overnight bacterial culture using the DNeasy Blood and Tissue Kit (Qiagen, Hilden, Germany). The purity of the genomic DNA was assessed using a NanoDrop ND-1000 spectrophotometer (Thermo Fisher Scientific, Waltham, USA), and its quantification was performed using a Qubit 4.0 fluorometer (Invitrogen Life Technologies, Massachusetts, USA). DNA libraries were prepared using the Illumina DNA Prep kit (Illumina, California, USA) and sequenced on the Illumina MiSeq (Illumina, California, USA) system using the V3 600 cycles cartridge at the genomic centre of icddr,b. The sequence reads was trimmed and filtered using Trimmomatic 0.39 [79], and *de novo* assemblies of the reads were generated using SPAdes 3.11.1 [80]. The quality of the assemblies was assessed using QUAST v5.02 [81]. Prokka *de novo* [82] was used to annotate the genomes and the program was run in fast mode with the genus-specific BLAST database. The genome features and associated metadata are tabulated in Supplementary Table-S1.

### 4.10 WGS based molecular typing and genome visualization

The sequence types (STs) of the isolates were determined using the Achtman scheme based on the whole genome sequencing data [83]. The serotype and multiple locus sequence type of the isolates were determined using the SeroTypeFinder and MLST tools from the Center for Genomic Epidemiology, respectively [83,84]. Additionally, the core genome MLST (cgMLST) of the isolates was investigated using the EnteroBase server (https://enterobase.warwick.ac.uk/species/index/ecoli accessed on April 20, 2022), which employs the 2513 loci [85]. Moreover, EnteroBase was employed for in silico phylotype predictions following the Clermont scheme [86] and fimH allelic designations [85]. Ribosomal MLST, hierarchical cgMLST clustering, and wgMLST were further conducted with the core genome data in EnteroBase. RAST 2.0 server (https://rast.nmpdr.org/rast.cgi accessed on November 04, 2022) [87] was used to detect the size, GC content, average coverage, length, N50, L50, and RNAs of the isolates and predict the subsystems in the genome. Lastly, the Proksee server (https://proksee.ca/) [88] was employed to view the annotated genomes of the sequenced isolates and map the contigs of the isolates to the complete genome of EC-958.v1 (GCA_000285655.3) for visualization of their genomic organization.

### 4.11 Resistome and virulome profiling

The ARGs and virulence factors of the *E. coli* genomes were identified using the ABRicate v.1.0.1 [89] utilizing the ARG-ANNOT [90] and VFDB databases [91]. Further, the pathogenic human potential of the isolates was investigated using the pathogenicity prediction web-server named PathogenFinder with isolates harbouring a threshold of ≥90% being identified as human pathogens [92].

### 4.12 Mobile genetic elements

The detection of plasmid replicons and the plasmid incompatibility groups were detected using the PlasmidFinder 2.1 (https://cge.food.dtu.dk/services/PlasmidFinder/accessed on 21 December 2022) database, and the sequence typing of the plasmid replicons was done using the pMLST 2.0 (https://cge.food.dtu.dk/services/pMLST/accessed on 21 December 2022) database [93]. Insertion sequences (IS) were detected in silico using MGEfinder, which investigates the synteny of virulence determinants and antibiotic resistant genes with mobile genetic elements (MGE) [94]. The PHAge Search Tool Enhanced Release (PHASTER) server was utilized to identify, annotate and visualize prophage sequences [95]. The putative CRISPR system and Cas cluster were assessed using CRISPRCasFinder (https://crisprcas.i2bc.paris-saclay.fr/CrisprCasFinder/Index accessed on 16 December 2022). The presence of tandem repeats was investigated using equicktandem (https://www.bioinformatics.nl/cgi-bin/emboss/equicktandem accessed on 10 December 2022) from EMBOSS software suite using default parameters [96].

### 4.13 Pangenome analysis

To get a better understanding of the similarity and the relationships among ESBL-producing *E. coli*, a pangenome analysis of the four sequenced genomes and 46 genome assemblies originating from Bangladesh from NCBI (https://www.ncbi.nlm.nih.gov/assembly) was performed. Prokka V1.13 [82] was used to annotate the assembled genomes. The number of core and accessory genes present in the genomes of the isolates was determined by the pangenome analysis software Roary V3.20 [97]. Polymorphic sites were extracted from the Roary output file using SNP-sites. The phylogenetic tree was constructed in IQ tree [98], and the tree was visualized with Phandango [99] and ggtree V3.17 [100].

## Funding

This work was supported by the 10.13039/100000030Centers for Disease Control and Prevention (CDC), grant number CoAg#5U01GH001207.

## Author contribution statement

Zahid Hayat Mahmud, M. Moniruzzaman, Mohammed Tanveer Hussain, Monir Hossain, Mohammad Atique Ul Alam, Md. Sakib Hossain, Md. Tamzid Islam and Faisal Chowdhury Galib: Conceived and designed the experiments. M. Moniruzzaman, Mohammed Tanveer Hussain, Sobur Ali, Monir Hossain and Partha Paul: Performed the experiments. M. Moniruzzaman, Mohammed Tanveer Hussain, Faisal Chowdhury Galib, Md. Tamzid Islam, Sobur Ali, Monir Hossain, Md. Sakib Hossain, Mohammad Atique Ul Alam, Partha Paul, Zahid Hayat Mahmud, Mahbubul Hasan Siddiqee: Analyzed and interpreted the data. Zahid Hayat Mahmud, Md. Shafiqul Islam, Dinesh Mondal, Shahana Parveen and Mahbubul Hasan Siddiqee: Contributed reagents, materials, analysis tools or data. Mohammed Tanveer Hussain, M. Monirruzzaman, Sobur Ali, Md. Sakib Hossain, Mohammad Atique Ul Alam, Monir Hossain and Zahid Hayat Mahmud: Wrote the paper.

## Data availability statement

Data will be made available on request.

## Declaration of competing interest

The authors declare that they have no known competing financial interests or personal relationships that could have appeared to influence the work reported in this paper.

## Figures and Tables

**figs1:**