# Supplementary material for: Multidrug-resistant Escherichia coli isolated from patients and surrounding hospital environments in Bangladesh: A molecular approach for the determination of pathogenicity and resistance
Source: Heliyon. 2023 Nov 7;9(11):e22109. doi: 10.1016/j.heliyon.2023.e22109 (PMC10679508; doi:10.1016/j.heliyon.2023.e22109)
Supplement: Multimedia component 1 [file mmc1.docx]

**Supplementary Table-S1-Summary of genotypic features of ESBL *E. coli***

| **ID** | **GC Content** | **N50** | **L50** | **No. Contigs** | **rMLST*** | **wgMLST** | **Coverage** | **Length** | **RNA** |
| --- | --- | --- | --- | --- | --- | --- | --- | --- | --- |
| LEH-23 | 50.8 | 140881 | 12 | 183 | 14948 | 260077 | 102 | 5088726 | 89 |
| LEH-24 | 50.61 | 214537 | 8 | 50 | 30298 | 253729 | 113 | 4780012 | 82 |
| LEH-60 | 50.79 | 140881 | 13 | 181 | 14948 | 253730 | 86 | 5102304 | 89 |
| LEH-147 | 50.69 | 106447 | 12 | 152 | 40960 | 253733 | 167 | 5161969 | 88 |
|  | GC: Guanine-Cytosine content, rMLST: ribosomal Multilocus Sequence Type, wgMLST: Whole genome Multilocus Sequence Type, RNA: Ribonucleic acid | | | | | | | |  |
