# Supplementary material for: Multidrug-resistant Escherichia coli isolated from patients and surrounding hospital environments in Bangladesh: A molecular approach for the determination of pathogenicity and resistance
Source: Heliyon. 2023 Nov 7;9(11):e22109. doi: 10.1016/j.heliyon.2023.e22109 (PMC10679508; doi:10.1016/j.heliyon.2023.e22109)
Supplement: Multimedia component 2 [file mmc2.docx]

**Supplementary Material**

Table S2: Primer sequences and amplicon sizes for antibiotic-resistance genes, virulent genes and their associated pathotypes

|  | **Target**  **gene** | **Primer**  **name** | | **Sequence**  **(5’ 🡪 3’)** | **Product**  **size (bp)** | **Reference** |
| --- | --- | --- | --- | --- | --- | --- |
| **ESBL primers** | *bla_SHV_* | SHV-F | | CTTTATCGGCCCTCACTCAA | 237 | [1] |
|  |  | SHV-R | | AGGTGCTCATCATGGGAAAG |  |  |
|  | *bla_TEM_* | TEM-F | | CGCCGCATACACTATTCTCAGAATGA | 445 | [1] |
|  |  | TEM-R | | ACGCTCACCGGCTCCAGATTTAT |  |  |
|  | *bla_CTX-M_* | CTX-M-F | | ATGTGCAGYACCAGTAARGTKATGGC | 593 | [1] |
|  |  | CTX-M-R | | TGGGTRAARTARGTSACCAGAAYCAGCGG |  |  |
|  | *bla_OXA_* | OXA-F | | ACACAATACATATCAACTTCGC | 813 | [1] |
|  |  | OXA-R | | AGTGTGTTTAGAATGGTGATC |  |  |
|  | *bla_CTX-M-1_* group | M-13-Upper | | GGTTAAAAAATCACTGCGTC | 866 | [2] |
|  |  | M-13-Lower | | TTGGTGACGATTTTAGCCGC |  |  |
|  | *bla_CTX-M-9_* group | M-9-Upper | | ATGGTGACAAAGAGAGTGCA | 870 | [3] |
|  |  | M-9-Lower | | CCCTTCGGCGATGATTCTC |  |  |
|  | *bla_CTX-M-2_* group | M25-upper | | ATGATGACTCAGAGCATTCG | 866 | [3] |
|  |  | M25-lower | | TGGGTT ACGATTTTCGCCGC |  |  |
|  | *bla_CTX-M-8_* group | CTX-M-8-F | | TCGCGTTAAGCGGATGATGC | 688 | [3] |
|  |  | CTX-M-8-F | | AACCCACGATGTGGGTAGC |  |  |
|  | *bla_NDM-1_* | NDM-F | | CTTCCAACGGTTTGATCGTC | 465 | [4] |
|  |  | NDM-R | | TAGTGCTCAGTGTCGGCATC |  |  |
| **Intestinal pathogenic primers** | *estA* | ST-F | | GCTAAACCAGTA^G^_A_GGTCTTCAAAA | 147 | [5] |
|  |  | ST-R | | CCCGGTACA^G^_A_GCAGGATTACAACA |  |  |
|  | *eltB* | LT-F | | CACACGGAGCTCCTCAGT C | 508 | [6] |
|  |  | LT-R | | CCCCCAGCCTAGCTTAGTTT |  |  |
|  | *bfpA* | bfpA-F | | GGAAGTCAAATTCATGGGGG | 300 | [6] |
|  |  | bfpA-R | | GGAATCAGACGCAGACTGGT |  |  |
|  | *eae* | eae-F | | CCCGAATTCGGCACAAGCATAAGC | 881 | [7] |
|  |  | eae-R | | CCCGGATCCGTCTCGCCAGTATTCG |  |  |
|  | *aaiC* | aaiC-F | | ATTGTCCTCAGGCATTTCAC | 215 | [6] |
|  |  | aaiC-R | | ACGACACCCCTGATAAACAA |  |  |
|  | *aat* | _p_cvd432-F | | CTGGCGAAAGACTGTATCAT | 650 | [8] |
|  |  | _p_cvd432-R | | CAATGTATAGAAATCCGCTGTT |  |  |
|  | *iaa* | ial upper | | CTGGATGGTATGGTGAGG | 320 | [9] |
|  |  | ial lower | | GGAGGCCAACAATTATTTCC |  |  |
|  | *ipaH* | Shig-1 | | TGGAAAAACTCAGTGCCTCT | 424 | [10] |
|  |  | Shig-2 | | CCAGTCCGTAAATTCATTCT |  |  |
|  | *stx1* | stx1F | | CACAATCAGGCGTCGCCAGCGCACTTGCT | 606 | [11] |
|  |  | stx1R | | TGTTGCAGGGATCAGTGGTACGGGGATGC |  |  |
|  | *stx2* | stx2F | | CCACATCGGTGTCTGTTATTAACCACACC | 372 | [12] |
|  |  | stx2R | | GCAGAACTGCTCTGGATGCATCTCTGGTC |  |  |
| **ExPEC primers** | *focG* | focG_106F | | CGTACCTGTACCATTGGTAATGGAGG | 366 | [13] |
|  |  | focG_471R | | TGAATTAATACTTCCCGCACCAGC |  |  |
|  | *kpsMII* | kpsMII_121F | | GCGCATTTGCTGATACTGTTG | 452 |  |
|  |  | kpsMII_572 | | GGGAACATGATGCAGGAGATG |  |  |
|  | *papA* | papA_67F | | ATGGCAGTGGTGTCTTTTGGTG | 717 |  |
|  |  | papA_+202R | | CGTCCCACCATACGTGCTCTTC |  |  |
|  | *sfaS* | sfaS_210F | | GTCTCTCACCGGATGCCAGAATAT | 138 |  |
|  |  | sfaS_347R | | GCATTACTTCCATCCCTGTCCTG |  |  |
|  | *afa* | afa F | | GGCAGAGGGCCGGCAACAGGC | 594 |  |
|  |  | afa R | | CCCGTAACGCGCCAGCATCTC |  |  |
|  | *hlyD* | hlyD_92F | | CTCCGGTACGTGAAAAGGAC | 904 |  |
|  |  | hlyD_995R | | GCCCTGATTACTGAAGCCTG |  |  |
|  | *iutA* | iutA_674F | | ATCGGCTGGACATCATGGGAAC | 314 |  |
|  |  | iutA_987R | | CGCATTTACCGTCGGGAACGG |  |  |
| **ERIC Primer** | ERIC | ERIC-2 | | AAGTAAGTGACTGGGGTGAGCG | N/A | [3] |
| **Gene** | | | **Pathotype** | | | |
| *estA* | | | ETEC | | | |
| *eltB* | | |  |  |  |  |
| *bfpA* | | | EPEC | | | |
| *eae* | | |  |  |  |  |
| *aaiC* | | | EAEC | | | |
| *aat* | | |  |  |  |  |
| *iaa* | | | EIEC | | | |
| *ipaH* | | |  |  |  |  |
| *stx1* | | | EHEC | | | |
| *stx2* | | |  |  |  |  |

**References:**

[1] H. Fang, F. Ataker, G. Hedin, K. Dornbusch, Molecular epidemiology of extended-spectrum β-lactamases among Escherichia coli isolates collected in a Swedish hospital and its associated health care facilities from 2001 to 2006, J. Clin. Microbiol. 46 (2008) 707–712. https://doi.org/10.1128/JCM.01943-07.

[2] N. Guessennd, S. Bremont, V. Gbonon, A. Kacou-Ndouba, E. Ekaza, T. Lambert, M. Dosso, P. Courvalin, Qnr-type quinolone resistance in extended-spectrum beta-lactamase producing enterobacteria in Abidjan, Ivory Coast, Pathol. Biol. (Paris). 56 (2008) 439–446.

[3] M.S. Hossain, S. Ali, M. Hossain, S.Z. Uddin, M. Moniruzzaman, M.R. Islam, A.M. Shohael, M.S. Islam, T.H. Ananya, M.M. Rahman, M.A. Rahman, M. Worth, D. Mondal, Z.H. Mahmud, ESBL Producing Escherichia coli in Faecal Sludge Treatment Plants: An Invisible Threat to Public Health in Rohingya Camps, Cox’s Bazar, Bangladesh, Front. Public Heal. 9 (2021). https://doi.org/10.3389/fpubh.2021.783019.

[4] M.A. Islam, P.K. Talukdar, A. Hoque, M. Huq, A. Nabi, D. Ahmed, K.A. Talukder, M.A.C. Pietroni, J.P. Hays, A. Cravioto, Emergence of multidrug-resistant NDM-1-producing Gram-negative bacteria in Bangladesh, Eur. J. Clin. Microbiol. Infect. Dis. 31 (2012) 2593–2600.

[5] T.V. Nguyen, P. Le Van, C. Le Huy, K.N. Gia, A. Weintraub, Detection and characterization of diarrheagenic Escherichia coli from young children in Hanoi, Vietnam, J. Clin. Microbiol. 43 (2005) 755–760.

[6] P.K. Talukdar, M. Rahman, M. Rahman, A. Nabi, Z. Islam, M.M. Hoque, H.P. Endtz, M.A. Islam, Antimicrobial resistance, virulence factors and genetic diversity of Escherichia coli isolates from household water supply in Dhaka, Bangladesh, PLoS One. 8 (2013) e61090.

[7] E. Oswald, H. Schmidt, S. Morabito, H. Karch, O. Marches, A. Caprioli, Typing of intimin genes in human and animal enterohemorrhagic and enteropathogenic Escherichia coli: characterization of a new intimin variant, Infect. Immun. 68 (2000) 64–71.

[8] J.A. Mohammed, H.L. DuPont, Z.-D. Jiang, J. Flores, L.G. Carlin, J. Belkind-Gerson, F.G. Martinez-Sandoval, D. Guo, A.C. White Jr, P.C. Okhuysen, A single-nucleotide polymorphism in the gene encoding osteoprotegerin, an anti-inflammatory protein produced in response to infection with diarrheagenic Escherichia coli, is associated with an increased risk of nonsecretory bacterial diarrhea in North Ame, J. Infect. Dis. 199 (2009) 477–485.

[9] G. Frankel, J.A. Giron, J. Valmassoi, G.K. Schoolnik, Multi‐gene amplification: simultaneous detection of three virulence genes in diarrhoeal stool, Mol. Microbiol. 3 (1989) 1729–1734.

[10] D. Lüscher, M. Altwegg, Detection of shigellae, enteroinvasive and enterotoxigenic Escherichia coli using the polymerase chain reaction (PCR) in patients returning from tropical countries, Mol. Cell. Probes. 8 (1994) 285–290.

[11] M.A. Islam, A.E. Heuvelink, E. De Boer, P.D. Sturm, R.R. Beumer, M.H. Zwietering, A.S.G. Faruque, R. Haque, D.A. Sack, K.A. Talukder, Shiga toxin-producing Escherichia coli isolated from patients with diarrhoea in Bangladesh, J. Med. Microbiol. 56 (2007) 380–385.

[12] A.E. Heuvelink, N. Van de Kar, J. Meis, L.A.H. Monnens, W.J.G. Melchers, Characterization of verocytotoxin-producing Escherichia coli O157 isolates from patients with haemolytic uraemic syndrome in Western Europe, Epidemiol. Infect. 115 (1995) 1–3.

[13] E. Franz, C. Veenman, A.H.A.M. Van Hoek, A. de Roda Husman, H. Blaak, Pathogenic Escherichia coli producing Extended-Spectrum β-Lactamases isolated from surface water and wastewater, Sci. Rep. 5 (2015) 1–9.
